# Uncovering protein conformational dynamics within two‐component viral biomolecular condensates

**DOI:** 10.1002/pro.70181

**Published:** 2025-06-16

**Authors:** Alice Colyer, Julia Acker, Alexander Borodavka, Antonio N. Calabrese

**Affiliations:** ^1^ Astbury Centre for Structural Molecular Biology, School of Molecular and Cellular Biology, Faculty of Biological Sciences University of Leeds Leeds UK; ^2^ Department of Chemical Engineering and Biotechnology University of Cambridge Cambridge UK

**Keywords:** biomolecular condensates, hydrogen–deuterium exchange mass spectrometry, native mass spectrometry, protein dynamics, rotavirus

## Abstract

Biomolecular condensates selectively compartmentalize and organize biomolecules within the crowded cellular milieu and are instrumental in some disease mechanisms. Upon infection, many RNA viruses form biomolecular condensates that are often referred to as viral factories. The assembly mechanism of these viral factories remains poorly defined but involves transient, non‐stoichiometric protein/RNA interactions, making their structural characterization challenging. Here, we sought to investigate the structural dynamics and intermolecular interactions of the key proteins responsible for condensate formation upon rotavirus infection, namely NSP2 (an RNA chaperone) and NSP5 (an intrinsically disordered protein [IDP]), using a combination of hydrogen–deuterium exchange mass spectrometry (HDX‐MS), native MS, and biophysical tools. Our data reveal key structural features of intrinsically disordered NSP5 that are vital for condensate assembly and highlight inter/intra‐protein interactions involved in condensate assembly. Moreover, we demonstrate that within a condensate there are altered conformational dynamics within the C‐terminal region of NSP2, which has previously been shown to play a role in regulating its RNA chaperoning activity, and in the disordered regions of NSP5. We propose that altered conformational dynamics in NSP2 and NSP5 are critical for regulation of RNA annealing within a biomolecular condensate and for condensate assembly/client recruitment, respectively. Combined, our data demonstrate that the unique environment within a biomolecular condensate can tune functionally important protein conformational dynamics, which may play a crucial role in the replication of rotaviruses.

## 1 INTRODUCTION

Biomolecular condensates are utilized within crowded cellular environments to selectively compartmentalize and organize biomolecules, facilitating a variety of cellular processes and disease mechanisms, including RNA metabolism, neurodegeneration, and replication of RNA viruses (Conicella et al., 2020; Geiger et al., 2021; Hnisz et al., 2017). The formation of biomolecular condensates is often described as occurring via liquid–liquid phase separation (LLPS), which dictates that the assembly of condensates is mediated by a complex network of weak, multivalent homo‐ and/or heterotypic non‐covalent interactions (Kilgore & Young, 2022). Typically, condensate formation is driven by protein molecular scaffolds, with many protein drivers of condensate assembly possessing regions of intrinsic disorder or low sequence complexity (DiRusso et al., 2022). The “sticker and spacer” model is frequently used to describe the sequence determinants for the formation of biomolecular condensates (Choi et al., 2020). Within IDPs/IDRs, certain residues or small groups of residues known as “stickers” may be present that possess clustered charged, polar, or hydrophobic residues (Holehouse et al., 2021). These regions subsequently form non‐covalent interactions with other “sticker” regions of the same or different proteins. “Sticker” regions are separated by “spacer” regions, which are often enriched in charged residues (Bremer et al., 2022). Moreover, the formation of biomolecular condensates can be driven by both disordered and folded domains within proteins. For example, folded SH3 domains involved in cellular signaling are instrumental in the formation of biomolecular condensates specifically by interacting with proline‐rich motifs (Li et al., 2012). Indeed, the protein Grb2, which has SH3 domains, and Sos1, which has several proline‐rich motifs, coordinate the assembly of biomolecular condensates (Shin and Brangwynne, 2017). Likewise, the P‐granule protein, PGL‐1, must be folded to facilitate mRNA binding and subsequent biomolecular condensate formation (Schmidt et al., 2021).

Whether predominantly ordered or disordered, the protein scaffolds of a condensate can selectively recruit client molecules (RNA/DNA or protein) into biomolecular condensates (Banani et al., 2016). The complex, heterogeneous, and transient nature of condensates makes it challenging to precisely characterize the biomolecular interactions driving condensate formation, including the interactions between scaffold molecules driving condensate assembly and the intermolecular interactions driving client recruitment. Indeed, most of our knowledge of biomolecular condensate assembly mechanisms has been gained by studying isolated single‐protein systems in vitro (Chatterjee et al., 2022; Ingólfsson et al., 2023), and information is more sparse for complex multiprotein systems where heterotypic interactions between scaffold molecules are key to condensate assembly (Banani et al., 2017).

**FIGURE 1:**
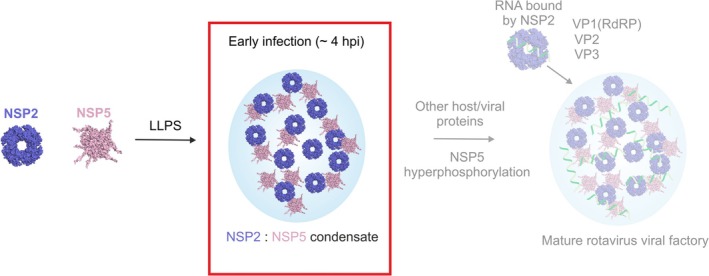
Biomolecular condensate formation driven by NSP2 and NSP5. NSP2 and NSP5 interact at low micromolar concentrations in vitro and form biomolecular condensates in RV‐infected cells at approximately 2–4 h post‐infection (hpi) (red box). Progression of viral infection is associated with RNA enrichment in VFs, likely bound by the RNA chaperone NSP2, and phosphorylation of NSP5. These VFs accumulate the viral pre‐genomic ssRNA, along with other components of the viral replicative machinery, including the RNA Polymerase (VP1), the inner capsid protein VP2, the capping enzyme (VP3), and additional capsid‐forming proteins (for simplicity, not shown). Here, we have focused on investigating the interactions driving early biomolecular condensate formation by NSP2 and NSP5 (red box).

Growing evidence suggests that many viral factories (VFs) are biomolecular condensates that play pivotal roles in the replication and maturation of many RNA viruses, including SARS‐CoV‐2, rabies, and group A rotaviruses (RV) (Alenquer et al., 2019; Geiger et al., 2021; He et al., 2024; Nevers et al., 2022; Perdikari et al., 2020; Zhou et al., 2019). During RV infection, the key drivers of VF formation are NSP5, a 25 kDa IDP that has been suggested to form higher order oligomers (Martin et al., 2011), and the RNA chaperone NSP2 (Geiger et al., 2021), which forms a 302 kDa octameric assembly that has been studied by both x‐ray crystallography and cryo‐EM (Hu et al., 2012; Jiang et al., 2006). While there is some sequence variation (length and residues) in NSP2 and NSP5 across Group A rotaviruses, it has been shown that most variants are capable of forming VFs (Lee et al., 2024). RNA interference (RNAi) experiments have demonstrated that VF formation is unable to occur without expression of both NSP2 and NSP5 in infected cells (López et al., 2005). Similarly, expression of either NSP2 or NSP5 alone in cells does not result in the formation of structures resembling VFs, whereas co‐expression of NSP2 and NSP5 in the absence of other viral proteins results in the formation of VF‐like structures, providing evidence that, together, these two proteins provide the platform for subsequent VF assembly (Fabbretti et al., 1999). Thus far, it is understood that upon RV infection, NSP2 and NSP5 interact, forming biomolecular condensates at low micromolar concentrations (Geiger et al., 2021; Strauss et al., 2023) (Figure 1), but the interactions driving VF formation have remained elusive due to their complex and transient nature. This molecular scaffold acts to recruit viral RNA and the key viral/host proteins needed for virus replication. Throughout the later stages of infection, the condensates can change in morphology, which is thought to be as a result of NSP5 hyperphosphorylation, but the structural mechanism of client recruitment and condensate maturation is poorly understood (Eichwald et al., 2002; Sotelo et al., 2010).

Structural mass spectrometry (MS) methods, such as native MS and hydrogen–deuterium exchange MS (HDX‐MS), are indispensable tools to study the architecture of dynamic systems, including IDPs, membrane proteins, and large, heterogenous protein complexes (Beveridge & Calabrese, 2021). Native MS can be used to inform on the stoichiometry of protein assemblies, particularly for complex stoichiometries characterized by dynamic heterogeneity, and has been used previously to study mechanisms of condensate formation (Sahin et al., 2023). HDX‐MS is an extremely attractive yet underexplored tool to study the formation of biomolecular condensates, allowing analysis of protein(s) and protein complexes in solution by deuterium labelling under physiological conditions (Masson et al., 2019). In a traditional “bottom–up” HDX‐MS experiment, protein(s) of interest are incubated with deuterium, typically on the second to hour timescale, allowing labile amides within the protein backbone to exchange for deuterium. Quantification of deuterium incorporation at the peptide level by MS can inform on the solvent accessibility of backbone amides, in addition to their involvement in intra‐ or intermolecular hydrogen bonding (Vinciauskaite & Masson, 2023).

Herein, we deployed an in vitro integrative structural MS approach to study the interactions driving biomolecular condensate formation by NSP2 and NSP5 and the conformational dynamics of NSP2/NSP5. Our study reveals that the C‐terminal region of NSP5 is essential for driving oligomerization of NSP5 to form a stable decamer, and without this region, condensate formation by NSP2 and NSP5 is ablated. Additionally, we show that the conformational dynamics of the C‐terminal helix of NSP2 are altered within NSP2/NSP5 biomolecular condensates. NSP2 is proposed to play a role in recruiting RNA into VFs for packaging and dsRNA synthesis (Bravo et al., 2018; Bravo et al., 2021; Taraporewala & Patton, 2004), and this C‐terminal helix plays an essential role in regulating this process. Altered conformational dynamics in this region when NSP2/NSP5 form a condensate suggest a role for the condensate environment in tuning NSP2 conformational dynamics and potentially the RNA chaperoning function of NSP2. The conformational dynamics of NSP5 are also altered within a bimolecular condensate. Together, our data are consistent with a model whereby structural transitions occur in NSP2 and NSP5 within a condensate that are key to their function, which has implications for understanding VF maturation and the initial steps underlying the formation of VFs.

## 2 RESULTS

### 2.1 Structured regions of NSP5 are vital for condensate assembly

**FIGURE 2:**
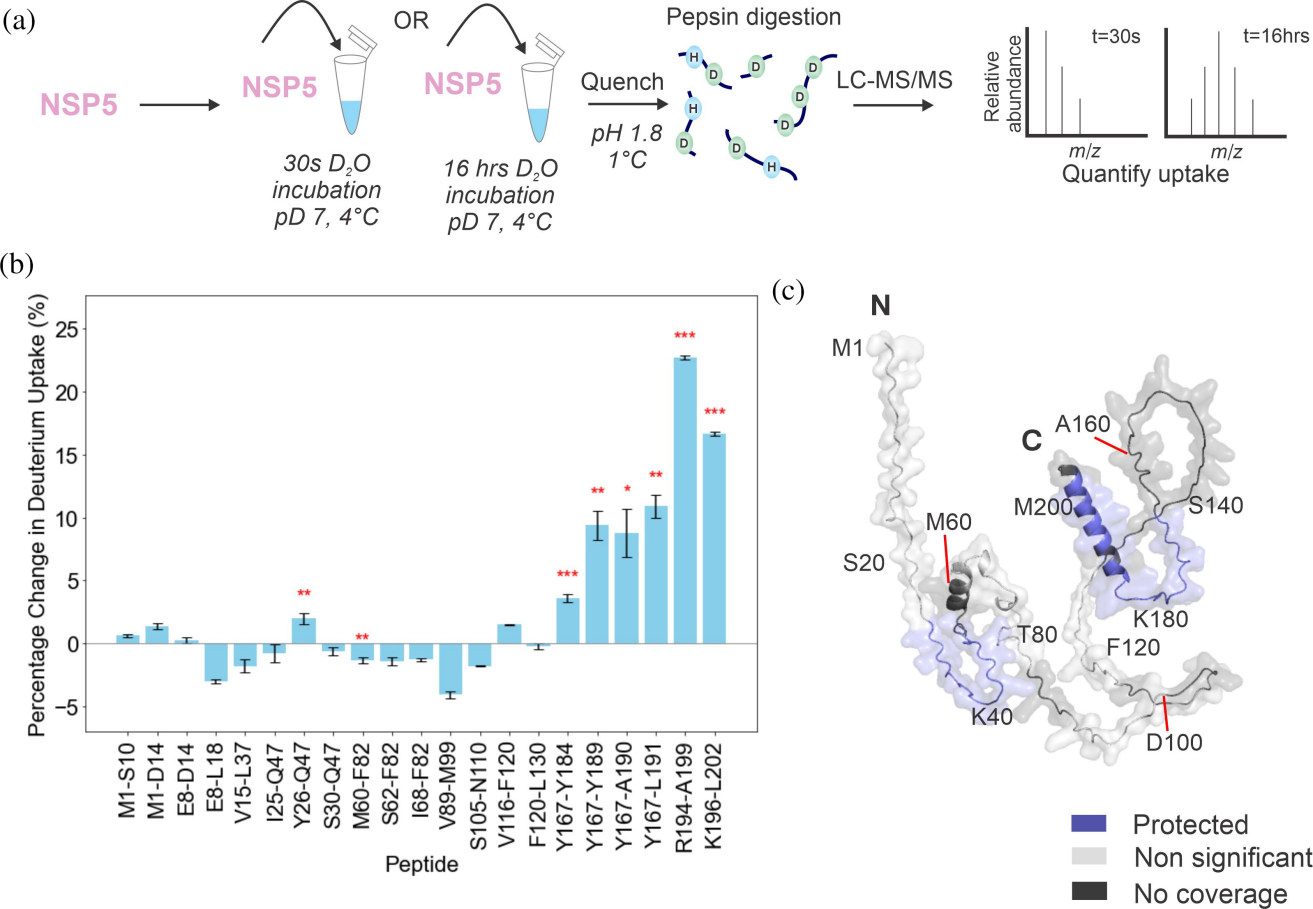
Identifying structured regions in the IDP NSP5 using HDX‐MS. (a) Experimental schematic. NSP5 was incubated with deuterium for either 30 s or 16 h before quenching of the exchange reaction by lowering the pH and temperature (see Methods). Digestion with pepsin followed by LC–MS/MS enabled the mass increase from deuterium incorporation to be quantified at the peptide level. (b) The percentage change in deuterium uptake for each peptide was calculated by dividing the difference in deuterium uptake (between 30 s and 16 h for each peptide) by the maximum deuterium uptake for each peptide. The standard error was propagated to account for uncertainty with uptake measurements. A *t*‐test was performed to identify statistically significant differences in uptake for each peptide (*p < 0.05, **p < 0.02, and ***p < 0.01). (c) AlphaFold2 model of monomeric NSP5 with protected regions (where there is a significant increase in deuterium uptake at 16 h vs. 30 s labelling) in blue. Regions where there was no significant increase in deuterium uptake at 16 h are shown in light gray and regions of no coverage are shown in dark gray. For clarity, the position of every twentieth residue is labeled in the chain.

While IDPs are typically thought of as key drivers of condensate formation, structured domains embedded within proteins that contain disordered regions also play a key role (Tibble & Gross, 2023). As NSP5 is predominantly an IDP and is employed as one of the two scaffold proteins required for rotavirus VF assembly, we utilized HDX‐MS to identify protected regions of NSP5, hypothesizing that these would correspond to regions of (partial) structure, using a strategy we have previously described (Minshull et al., 2024). Briefly, we compared the extent of deuterium uptake between short and long deuterium‐incubation time points (30 s and 16 h) (Figure 2a), rationalizing that backbone amides which are buried, and/or involved in intra‐ or intermolecular hydrogen bonding will take longer to reach maximum deuterium exchange compared to solvent exposed amides and/or those that are not involved in hydrogen bonds.

A total of 21 NSP5‐derived peptides were identified after digestion with pepsin, representing over 66% sequence coverage (Supplementary Figure 1a). We identify that in peptides derived from the 40 residues at the C‐terminus of NSP5 (spanning residues 167–191 and 194–202), there appears to be protection from deuterium exchange, suggesting this C‐terminal region of NSP5 is likely to possess partial structure (Figure 2b and Supplementary Figure 1b,c). This observation is supported by the AlphaFold2 model, which depicts the C‐terminal region of monomeric NSP5 to possess a structured α‐helix (Figure 2c). In all but one of the peptides from the first 130 residues of NSP5, maximum exchange was achieved at 30 s (Figure 2b and Supplementary Figure 1b,c), suggesting that this region of NSP5 lacks strong inter‐ or intramolecular hydrogen bonding interactions. This is consistent with the structural model of monomeric NSP5 derived from AlphaFold2 (Figure 2c), which predicts that the N‐terminus is predominantly disordered. We do observe protection from exchange in a peptide spanning residues 26–46 within the N‐terminal region, which could suggest the presence of partial structure in this region of the protein. Interestingly, this region has been proposed previously to be involved in NSP2 binding (Martin et al., 2011).

**FIGURE 3:**
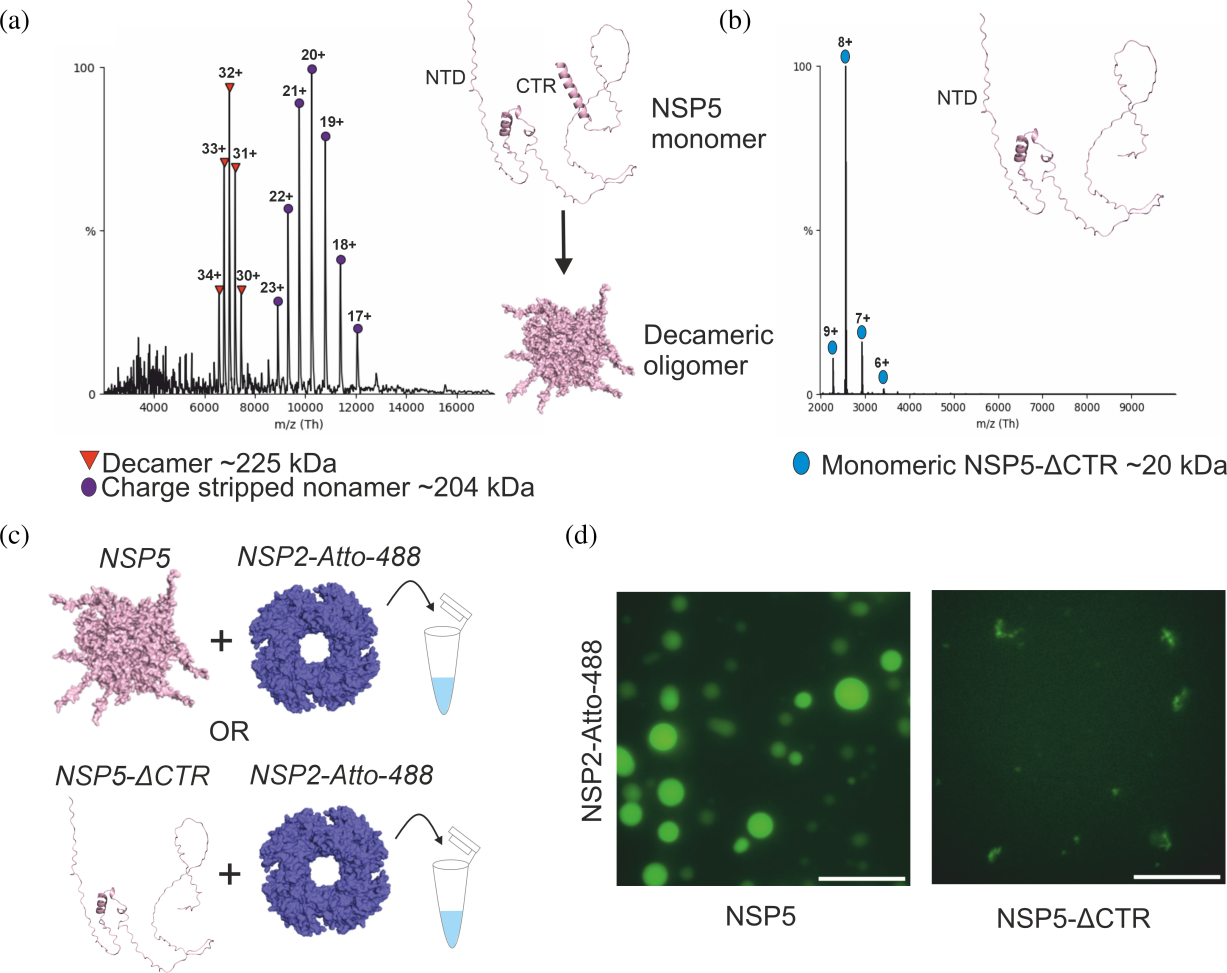
The C‐terminal region of NSP5 drives NSP5 oligomerization and is essential for biomolecular condensate formation by NSP2 and NSP5. (a) Native mass spectrum of NSP5. Red triangles represent the decameric assembly, while purple circles represent the charge‐stripped nonamer formed in the gas‐phase via collision‐induced dissociation (Belov et al., 2013). (b) Native mass spectrum of NSP5‐ΔCTR. Native MS experiments were performed using a ThermoFisher Q‐Exactive UHMR (see Methods). (c) Experimental schematic, demonstrating the method used to uncover the role of the NSP5‐CTR in biomolecular condensate formation driven by NSP2 and NSP5. NSP2 labeled with Atto‐488 was mixed with NSP5 or NSP5‐ΔCTR at 15 and 30 μM, respectively, and immediately imaged using an ONI Nanoimager S. (d) Wide‐field fluorescence microscopy images of spherical biomolecular condensates formed by NSP2 and NSP5 (scale bar = 10 μm, left panel). Removal of the CTR results in NSP5 that fails to form biomolecular condensates when mixed with NSP2 (right panel).

We, and others (Geiger et al., 2021; Martin et al., 2011), hypothesized that the C‐terminal region of NSP5 plays a role in mediating protein oligomerization; however, the precise oligomeric state adopted by NSP5 has remained ambiguous. Therefore, we utilized native MS to interrogate the oligomeric state of NSP5 and an NSP5 variant where the C‐terminal region (CTR) was removed (NSP5‐ΔCTR). While this approach means we are unable to uncover specific residues that could be involved in oligomerization, this does enable a conclusive evaluation of the role of the NSP5‐CTR in oligomer formation, guided by our HDX‐MS data, which identified a protected C‐terminus. In agreement with previous data from multi‐angle light scattering (Martin et al., 2011), the native mass spectrum of NSP5 is consistent with the protein forming a stable decamer (Figure 3a). Strikingly, in the mass spectrum of NSP5‐ΔCTR only monomeric NSP5 was detected, demonstrating that the CTR of NSP5 is essential for oligomerization, as removal prevents the formation of higher‐order assemblies (Figure 3a,b). Given our HDX‐MS data and the AlphaFold2 structural prediction, we sought to understand the role of the folded C‐terminal region in condensate formation, which revealed that removal of the NSP5‐CTR abrogates condensate formation when the protein is mixed with NSP2 (Figure 3c,d). Together, these data highlight the synergistic relationship between structure, oligomerization, and the ability of proteins to form condensates.

### 2.2 Elucidating protein structural dynamics within biomolecular condensates formed by NSP2 and NSP5

**FIGURE 4:**
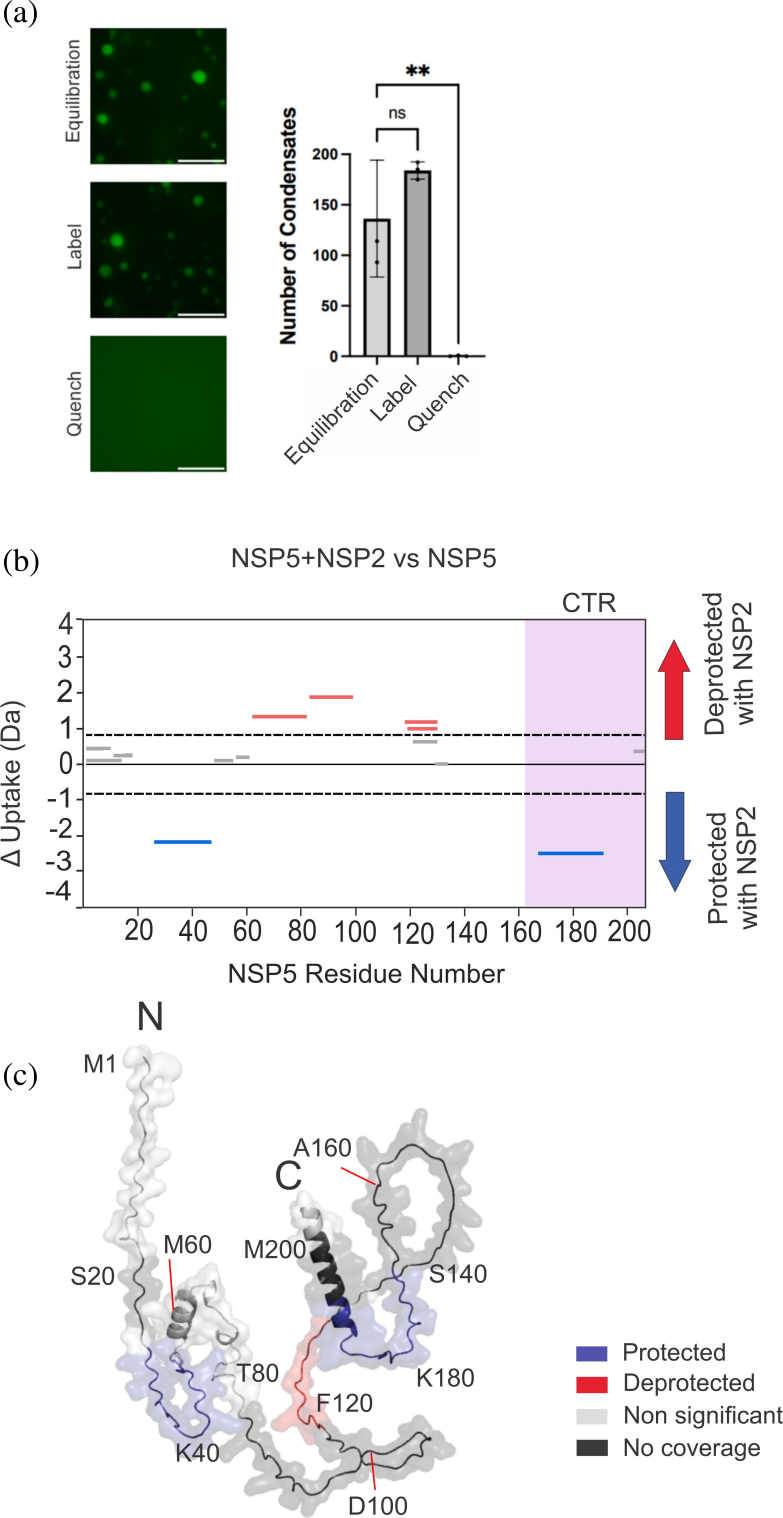
HDX‐MS analysis of NSP5 within a biomolecular condensate. (a) Fluorescence microscopy of NSP5 + NSP2‐Atto‐488 in equilibration (H_2_O‐containing), label (D_2_O‐containing), and quench HDX‐MS buffers. Biomolecular condensates form under deuterium labeling conditions and dissociate upon quenching of the HDX reaction (scale bar = 10 μm) (left). Quantification of the number of condensates per field of view (right). (b) Cumulative Woods' plot showing the summed differences in deuterium incorporation over all timepoints. Peptides from NSP5 that were significantly protected and deprotected when incubated with deuterium in the presence of NSP2 are shown in blue and red, respectively (confidence interval of 98%, see Methods). Wood's plots were produced using Deuteros (Lau et al., 2019). (c) AlphaFold2 model of monomeric NSP5 with regions of protection (blue), deprotection (red), non‐significant peptides (light gray), and no coverage (dark gray), mapped on the proposed structure. For clarity, the position of every twentieth residue is labeled in the chain.

The formation of biomolecular condensates relies on protein concentration, solvent conditions, and inter‐/intra‐protein hydrogen bonding. Here we sought to interrogate condensate assembly using HDX‐MS, and thus first sought to establish that the labeling conditions used for the experiment will not perturb condensate formation. Fluorescence microscopy confirms that under HDX‐MS buffer conditions, mixtures of NSP2 and NSP5 form biomolecular condensates, that condensates dissociate under the quench conditions used in the HDX‐MS experiment, and that there appears to be no aggregate formation under these conditions (Figure 4a and Supplementary Figure 2). The fact that condensate formation is consistent with HDX‐MS buffer conditions and reversible upon quenching confirms that NSP2/NSP5 condensates are amenable for study by HDX‐MS.

HDX‐MS analysis was then performed to compare the extent of protection/deprotection from exchange in NSP5 in the presence and absence or NSP2 (i.e., in dispersed versus condensed phases). Sequence coverage of NSP5 obtained post assignment was 67.5% across 15 peptides with a redundancy of 1.28 (Supplementary Figure 3a). Under condensate forming conditions, in the presence of NSP2, there are two protected and four deprotected NSP5 peptides (Figure 4b). Regions of NSP5 that become protected upon incubation with NSP2 lie within the disordered N‐terminal region (residues 26–47) (Figure 4b,c and Supplementary Figure 3b) and in some of the proposed structured CTR of NSP5 (residues 167–191), in addition to a stretch of residues leading up to this region (Figure 4b,c and Supplementary Figure 3b). The regions where protection from exchange was observed may be involved in interactions that facilitate NSP5 oligomerization that are stabilized within a condensate (Figure 3) or be directly participating in intermolecular interactions with NSP2, facilitating condensate assembly. Remarkably, these two regions of protection (corresponding to N‐terminal protected residues 26–47 and C‐terminal protected residues 167–191) overlap with previous reports that have indicated a role for residues both in the N‐ and C‐terminal regions of NSP5 in mediating interactions with NSP2. Specifically, results from pulldown assays have suggested critical roles for residues 1–33 and 180–198 (Eichwald et al., 2004; Lee et al., 2024), suggesting that the protection from exchange observed is due to NSP2 binding. Therefore, by leveraging the power of HDX‐MS, we uncover relevant interactions critical for condensate formation in the context of the full‐length proteins. Interestingly, we observed deprotection within the disordered N‐terminus, indicating that this region is more solvent exposed/less inter/intra‐protein hydrogen bonded in a condensate. This could be suggestive of an allosteric perturbation in NSP5 conformational dynamics within a biomolecular condensate. Such an increase in solvent exposure/reduction in intra‐protein hydrogen bonding with an NSP2/NSP5 condensate could be important for NSP5's function as a molecular scaffold and for the recruitment of additional components into maturing biomolecular condensates during the course of RV infection.

**FIGURE 5:**
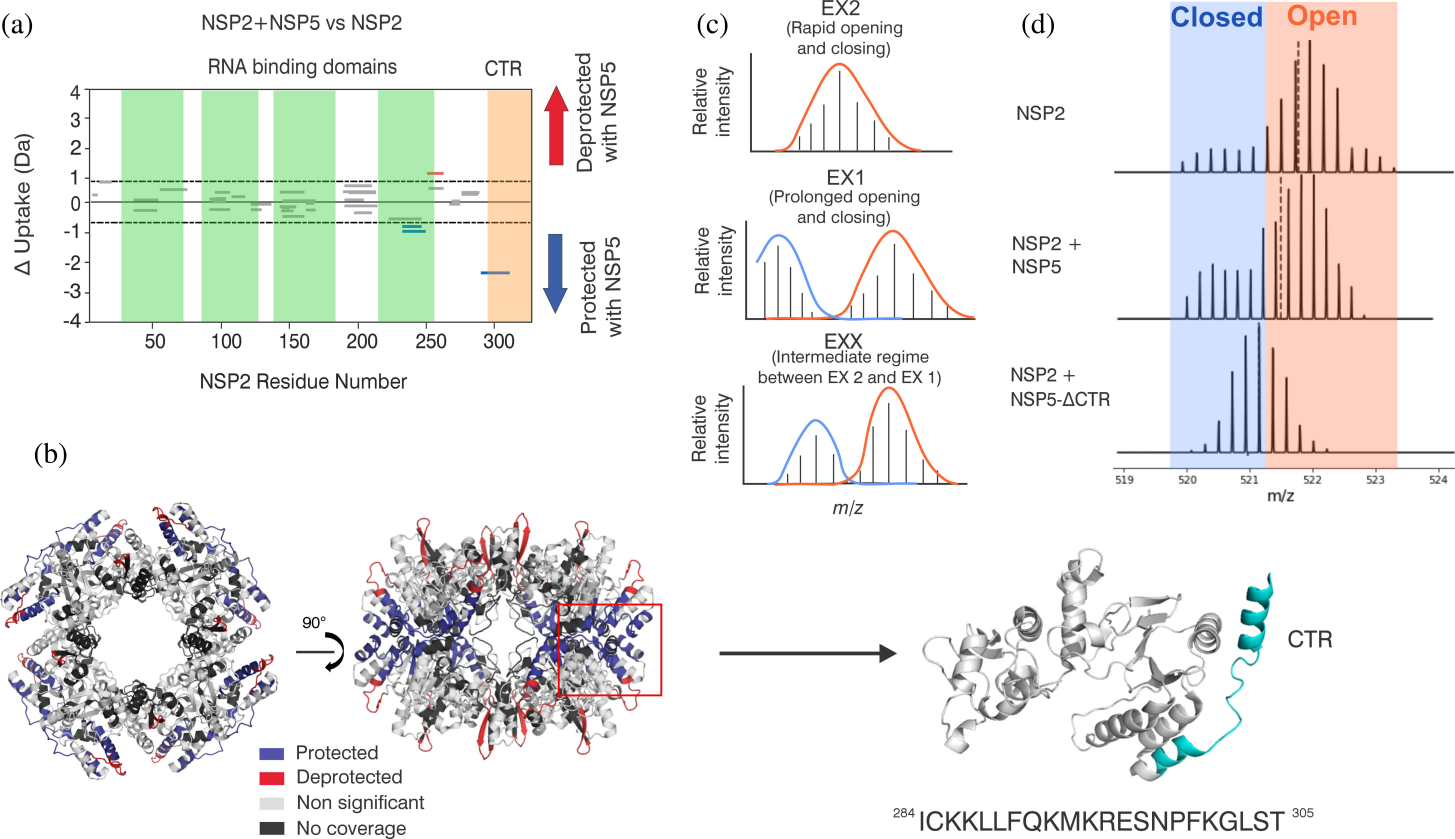
HDX‐MS analysis of NSP2 within a biomolecular condensate. (a) Cumulative Woods' plot showing the differences in deuterium incorporation over 0.5–10 min timepoints. Peptides from NSP2 that were significantly protected and deprotected when incubated with deuterium in the presence of NSP5 are shown in blue and red, respectively (confidence interval of 98%, see Methods). Produced using Deuteros (Lau et al., 2019). Green regions represent proposed RNA‐binding regions (Bravo et al., 2020), and the C‐terminal region (CTR) is represented in orange. (b) NSP2 octameric structure (PDB: IL9V) with regions of protection (blue), deprotection (red), non‐significant peptides (light gray), and no coverage (dark gray), mapped on the structure. A monomer subunit is indicated (red box). The structure of a monomeric subunit from NSP2 octamer is shown and CTR is highlighted in blue. The corresponding peptide sequence (residues 284–305) is shown below the monomeric structure. (c) Schematic of theoretical isotopic envelopes for EX2, EX1, and EXX kinetics detected by HDX‐MS. (d) Representative isotopic envelopes for a peptide spanning residues 284–305 of NSP2, for NSP2 alone and NSP2 + NSP5, and NSP2 + NSP5‐ΔCTR after incubation with D_2_O. Closed and open populations are represented by blue and orange boxes, respectively.

Next, we sought to identify changes in deuterium uptake in NSP2 within the condensates that form upon the addition of NSP5. A total of 72.7% coverage was obtained post assignment, represented by 39 peptides (Supplementary Figure 4a). Interestingly, we observe a deprotected peptide (residues 245–257) in addition to a protected peptide (residues 227–244) that overlaps with the RNA binding regions on NSP2 we have previously identified (Bravo et al., 2021) (Figure 5a). This suggests that there may be some conformational change at or around the RNA binding sites on NSP2 within the condensate. Such changes may be important for regulating the RNA chaperone function of NSP2 (Bravo et al., 2021). Furthermore, we observed significant protection from exchange mapped to the C‐terminal region of NSP2 at residues 284–305, spanning across the electropositive groove of NSP2 (Figure 5a,b and Supplementary Figure 4b,c). This is particularly interesting due to the proposed importance of the NSP2‐CTR for RNA annealing, chaperone recycling (Bravo et al., 2021), and VF growth and maturation (Criglar et al., 2014; Nichols et al., 2023). This observation suggests that NSP5 binding could either directly or indirectly be implicated in regulating the RNA chaperone activity of NSP2 within a condensate.

Intriguingly, upon examination of the protected NSP2 peptide spanning residues 284–305, we observed the presence of a bimodal isotopic distribution, which is unique from the typical deuterium exchange kinetics observed in most scenarios. The typical deuterium exchange process follows so‐called EX2 kinetics, where a gradual incorporation of deuterium into the backbone amides of a protein is observed because of rapid conversion (relative to the rate of deuterium exchange) between exchange‐incompetent and exchange‐competent states, and this manifests as a unimodal isotopic distribution (Hodge et al., 2020) (Figure 5c). In the case of NSP2, we observe that a peptide corresponding to residues 284–305 from the C‐terminal helix of NSP2 uniquely appears to exhibit a bimodal isotopic distribution, even in the absence of NSP5, which is characteristic of EX1 kinetic behavior (Figure 5c,d). EX1 exchange is typical of protein regions that undergo large conformational transitions, from a closed (exchange incompetent) to an open (exchange competent) state, more slowly than the rate of deuterium exchange (Hodge et al., 2020; Oganesyan et al., 2018; Wales et al., 2013; Xiao, 2005). The situation when deuterium incorporation by both EX1 and EX2 kinetic mechanisms is present is called the EXX regime (Figure 5c). In the presence of NSP5, the relative abundance of the “closed” (exchange incompetent) form appears to increase in intensity relative to the “‘open” (exchange competent) form. This could be because binding results in protection from exchange, resulting in more of the “closed” form being detected, or because binding is triggering a change in conformational dynamics in this region within a biomolecular condensate that favors the closed state (Figure 5c). Uncovering which scenario, or indeed if a mixture of the two scenarios is occurring, is a challenge. Nevertheless, these data suggest that within a biomolecular condensate, the interaction of NSP2 with NSP5 produces an effect on the dynamic behavior of the CTR of NSP2 (Figure 5d). This change in conformational dynamics could be important for the RNA chaperoning function of NSP2, where this CTR has been shown to play a critical role (Bravo et al., 2021). To decouple this effect and assess if this kinetic behavior within the NSP2 C‐terminal helix is unique to biomolecular condensate formation, we performed HDX‐MS of NSP2 in the presence of NSP5‐ΔCTR, which we have demonstrated does not form biomolecular condensates (Figure 3). This revealed significant protection to residues 284–305 of NSP2, indicating that NSP5 maintains some affinity with NSP2 residues 284–305 even in the absence of biomolecular condensate formation (Supplementary Figure 5a,b). Strikingly, when bound to NSP5‐ΔCTR, the isotopic distribution appears unimodal, characteristic of typical EX2 kinetic behavior (i.e., rapid opening and closing reflecting protein “breathing motions”) (Figure 5c,d). The unimodal distribution observed here lies in between the “closed” and “open” forms detected when NSP2 was both alone and within a NSP2/NSP5 condensate. This suggests that in the absence of condensate formation, residues 284–305 of NSP2 are likely to exhibit an alternative conformational state in the bound state. Together, this suggests that the EX1 kinetic behavior observed in the absence/presence of full‐length NSP5 (Figure 5c) may be a hallmark of conditions where NSP2 and NSP5 interact and form biomolecular condensates and could be suggestive of a dynamic conformational change within the C‐terminal region of NSP2 that promotes RNA chaperoning.

Together, our HDX‐MS data show that within a biomolecular condensate, regions of protection and deprotection are observed in both NSP2 and NSP5. Regions of deprotection in both proteins suggest that changes in conformational dynamics/hydrogen bonding arise within a biomolecular condensate. Protection in NSP2 residues 284–305 suggests that this region could be involved in stabilizing condensates formed by NSP2/NSP5. However, deciphering if protection from exchange arises from either occlusion of these protein regions from solvent because of intermolecular associations, or allosteric changes in dynamics/hydrogen‐bonding, remains a challenge and may require cross‐validation with complementary methods.

## 3 DISCUSSION

Elucidating the structure, interactions, and dynamics of proteins within biomolecular condensates remains a challenging endeavor. While NMR and single‐molecule FRET measurements have provided some insights into specific systems, these experiments are often conducted using simplistic models comprising only a single protein species (Galvanetto et al., 2023; Kim et al., 2021). This contrasts with our understanding of the mesoscale dynamics of condensates, which are relatively well characterized in vitro and in cell by deployment of established methods such as FRAP (Taylor et al., 2019; Zhang & Shen, 2023). Here, we show that changes in protein structural dynamics occur in the condensate environment compared to the dispersed phase and that these dynamic behaviors can be studied using HDX‐MS.

NSP2 residues 227–244 and 284–305 could be involved in binding to residues 26–47 and 167–191 of NSP5 upon the formation of biomolecular condensates, as represented by the protected peptides in Figures 4 and 5. In addition, we observed deprotection spanning residues 118–130 of NSP5, suggesting weakening of the intra‐protein hydrogen bonding network within NSP5. While this deprotective effect is relatively small, small changes in uptake are not unexpected. The crowded nature of biomolecular condensates, which are likely to contain thousands of NSP5 molecules, would suggest that any dynamic perturbations detected by HDX‐MS will be an averaged measurement across the population. Given the transient and multivalent nature of interactions responsible for condensate formation (Banani et al., 2017; Darzacq & Tjian, 2022), this dynamic population will be highly heterogeneous in nature, and it is therefore not surprising that changes in deuterium exchange (an ensemble‐averaged measurement) are small upon condensate formation. Nevertheless, the differences in deuterium exchange we have detected are statistically significant.

**FIGURE 6:**
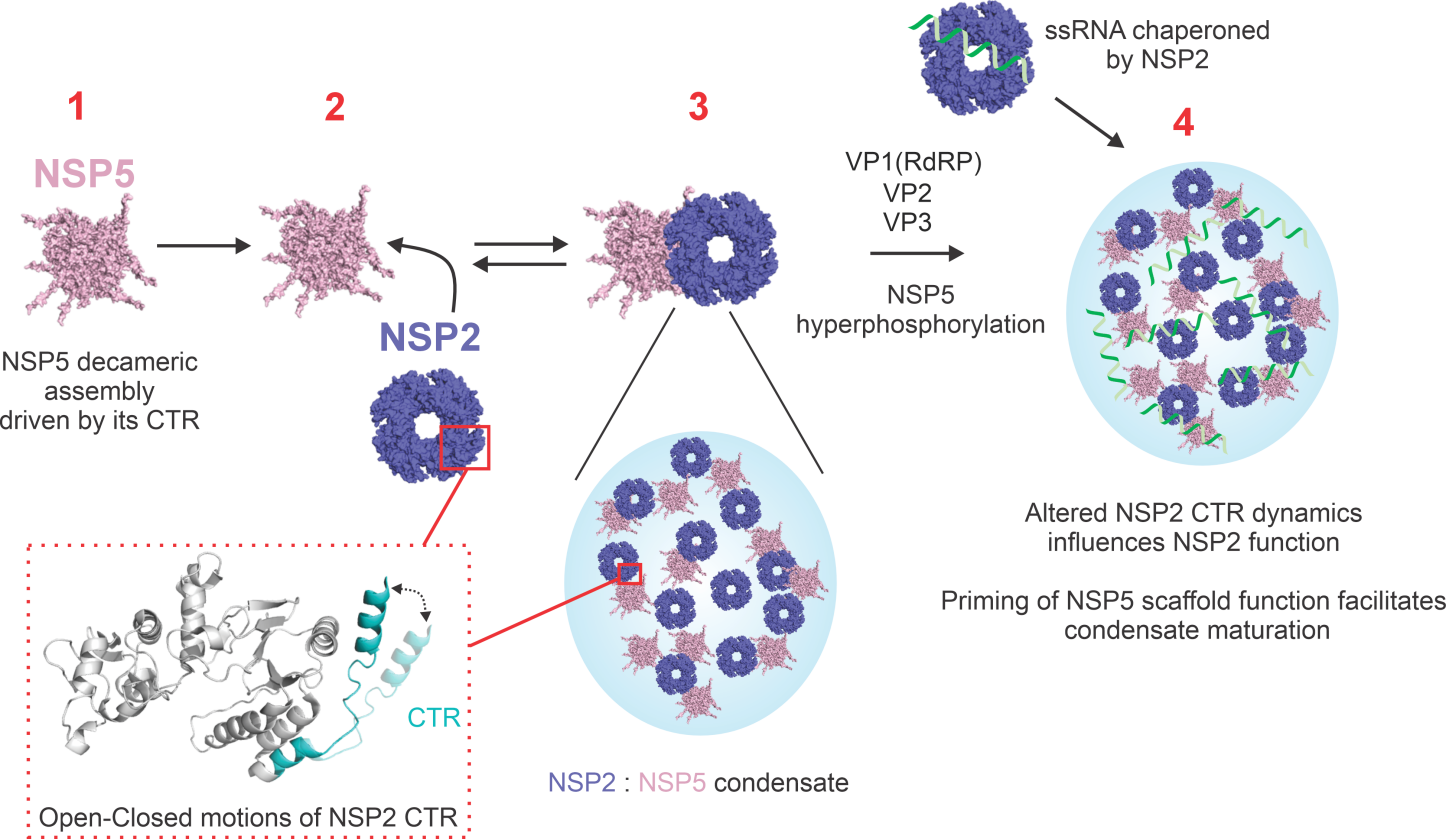
Proposed mechanism of biomolecular condensate formation by NSP2 and NSP5. (1) NSP5 assembles into a decameric oligomer driven by its CTR; (2) NSP5 recruits NSP2; (3) the proteins form biomolecular condensates. Binding of NSP5 within a biomolecular condensate induces a change in conformational dynamics of the NSP2 CTR, which may contribute to RNA dissociation from NSP2 or influence inter‐octamer interactions. (4) ssRNA transcripts are bound by VP1 and NSP2, and they partition into condensates along with VP2 and other capsid‐forming components. Opening (measured by deprotection in HDX‐MS) of the disordered regions of NSP5 facilitates client recruitment. During the late stages of infection, VFs undergo maturation that correlates with NSP5 hyperphosphorylation.

Intriguingly, we observe allosteric changes in dynamics in the IDP NSP5 within a condensate. While allostery is more closely associated with proteins possessing defined structured regions, reports of functionally important dynamic allostery in IDPs are now emerging (Berlow et al., 2018; Bhattarai & Emerson, 2020). The protection and deprotection from exchange we observed in NSP5 within a biomolecular condensate underscores the importance of NSP5 intra‐protein hydrogen bonding within a condensate. We hypothesize that the deprotection we have observed (i.e., weakening of intra‐protein hydrogen bonding) could help to reveal critical sequence motifs within NSP5 to facilitate its function as a molecular scaffold for client recruitment and mature viral factory assembly. Intriguingly, our data demonstrate that there is significant protection from exchange within the NSP2 C‐terminal helix, which has previously been identified by x‐ray crystallography to possess both an open and closed conformation responsible for inter‐octamer interactions, in addition to RNA binding and nucleoside triphosphate hydrolysis activity (Hu et al., 2012). Strikingly, residue K294 of NSP2 appears to be highly dynamic in the closed‐ to open‐state transition, experimentally confirming previous molecular dynamics simulations and reverse genetics studies of the K294E NSP2 mutant (Nichols et al., 2023). The kinetic behavior observed in our HDX‐MS experiments suggests that this region of NSP2 undergoes a specific allosteric structural change when incorporated in a condensate, or this region could be directly involved in forming “fuzzy” interactions with NSP5 that compete with NSP2‐RNA interactions (Tompa et al., 2015). We propose that a change in the structure/dynamics of this region within a biomolecular condensate may be relevant for NSP2‐mediated RNA annealing and release of bound RNA from NSP2. These observations combined with previous studies have enabled us to propose a model for NSP5/NSP2 condensate assembly and the role of protein structural dynamics of NSP2 within a condensate (Figure 6).

Molecular dynamics (MD) simulations, while beyond the scope of this manuscript, could represent a promising future avenue to further understand and validate the protein structural dynamics we have observed here by HDX‐MS within biomolecular condensates. For example, single‐molecule approaches used in conjunction with MD simulations have revealed the conformational distributions and rapid structural rearrangements of a number of IDPs involved in biomolecular condensate formation on the sub‐microsecond timescale (Galvanetto et al., 2023). Likewise, MD simulations of FUS (an RNA‐binding protein) revealed how molecular crowding influences protein dynamics within a biomolecular condensate, and how this shapes the macroscopic liquid‐like properties of condensates (Mukherjee & Schäfer, 2024). Furthermore, MD simulations have helped differentiate between “fast” and “slow” dynamics within biomolecular condensates from NMR data, demonstrating a kinetic interconversion between dilute and condensed phases, where increased intermolecular contacts under crowded environments correlate with faster dynamics (Guseva et al., 2023). While we have demonstrated here that HDX‐MS is able to capture peptide level resolution of the structural rearrangements within biomolecular condensates, we recognize that MD simulations could add complementary information that may aid in uncovering dynamic fluctuations in biomolecular condensates. Nevertheless, the work presented here demonstrates that within a biomolecular condensate formed by two different proteins, there are changes in the dynamic behavior of both folded and disordered regions. These changes in protein dynamics may play a critical role in facilitating condensate maturation and client recruitment, and in the case of NSP2, priming of its RNA annealing functions that are key to viral replication. While recent advanced cryo‐ET imaging can provide valuable insights into the components of viral replicative machinery and distinct structural states within biomolecular condensates (Goetz & Mahamid, 2020; Tollervey et al., 2023) (De Castro et al., 2013), it cannot capture the “fuzzy” interactions or the dynamic behaviors intrinsic to condensates formed through non‐stoichiometric interactions involving multiple intrinsically disordered proteins. Our findings underscore that uncovering these protein dynamics through structural proteomics approaches is critical for understanding the mechanisms driving condensate assembly and function.

## 4 MATERIALS AND METHODS

### 4.1 Expression and purification of NSP5 and NSP2

Recombinant N‐terminally Strep‐tagged NSP5 RV Strain A RF and NSP5‐ΔCTR were overexpressed in BL21‐DE3 Gold cells prepared in‐house as described by (Borodavka et al., 2017). A single colony was used to inoculate LB (100 mL) supplemented with 50 μg/mL kanamycin sulphate and incubated overnight at 37°C shaking at 200 rpm. 10 mL of the overnight starter culture was used to inoculate LB (1 L) supplemented with 1% glucose, 30 μM FeCl_3_, and 50 μg/mL kanamycin sulphate. The culture was incubated at 37°C with shaking at 200 rpm. When the culture reached an OD_600_ of 0.6, protein expression was induced by adding 1 mM IPTG, and the culture was incubated overnight at 24°C, shaking at 200 rpm. Cells were pelleted by centrifugation at 5000*g* for 20 min. The cell pellet was resuspended in lysis buffer (20 mM MOPS pH 7.1, 50 mM NaCl, 1 Complete Mini EDTA‐free protease inhibitor cocktail tablet per 50 mL cell lysate [Roche], 1 mM DTT), and the cells were lysed using a cell disruptor (Constant Flow Systems). The lysate was left at room temperature for 15 min before being supplemented with 10 μg/mL DNaseI and 10 mM MgCl_2_. The cell lysate was left to incubate at 37°C shaking at 280 rpm for 30 min and then centrifuged at 25,000*g* for 30 min at 4°C to isolate the inclusion bodies. The inclusion bodies were resuspended in resuspension buffer (20 mM MOPS pH 7.1, 50 mM NaCl, 1 mM DTT, 0.5% Triton‐X100) and centrifuged at 25,000*g* for 30 min at 4°C. A total of 2 inclusion body washes were performed with 20 mM MOPS pH 7.1, 50 mM NaCl, 1 mM DTT before pellets were stored at −20°C.

Inclusion bodies were solubilized in 50 mL solubilization buffer (6 M GuHCl, 20 mM MOPS pH 7.1, 1 mM DTT) for 1 h shaking at 250 rpm at 37°C. Insoluble material was removed by centrifugation at 3000*g* for 30 min. The supernatant was dialyzed overnight against 2 L of 20 mM MOPS pH 7.1, 10 mM β‐mercaptoethanol, 50 mM NaCl, 10 mM PMSF. A 5 mL StepTrap HP column (Cytiva) was equilibrated in 1 M NaCl, 20 mM MOPS pH 7.1. The supernatant was loaded onto the column and eluted over 5 column volumes of 1 M NaCl, 20 mM MOPS pH 7.1, 2 mM Desthiobiotin. Fractions were analyzed by SDS PAGE and peak fractions containing NSP5 were simultaneously concentrated and buffer exchanged into 20 mM MOPS pH 7.1 by centrifugal ultrafiltration.

Recombinant RV strain A RF C‐terminally His‐tagged NSP2 was overexpressed in BL21‐CodonPlus (DE3)‐RIL cells as per the manufacturer's protocol. A single colony was used to inoculate a starter culture of 100 mL LB supplemented with 1% glucose and 50 μg/mL kanamycin, and the culture was incubated overnight at 37°C with shaking at 200 rpm. 10 mL of overnight starter culture was used to inoculate a 400 mL subculture comprising LB supplemented with 1% glucose and 50 μg/mL kanamycin and incubated at 37°C shaking at 200 rpm until an OD_600_ of 0.6 was reached. Large‐scale 1 L cultures supplemented with 1% glucose and 50 μg/mL kanamycin were inoculated with 50 mL of the subculture and incubated at 37°C with shaking at 200 rpm until they reached an OD_600_ of 0.6. Protein expression was induced with 1 mM IPTG before overnight incubation at 24°C with shaking at 200 rpm. Cells were pelleted by centrifugation at 5000*g* for 20 min, immediately homogenized in lysis buffer (50 mM Tris–HCl pH 8.0, 300 mM NaCl, 1 Complete Mini EDTA‐free protease inhibitor cocktail tablet per 50 mL cell lysate [Roche]) and lysed using a cell disruptor (Constant Flow Systems) operating at 40 kpsi. 5 mM β‐mercaptoethanol, 10 mM MgCl_2_, and 1 mg/mL DNase were added to the lysate, and it was then incubated at room temperature for 15 min. The lysate was clarified by centrifugation (10,000*g*, 30 min, 4°C). The lysate was supplemented with 20 mM imidazole pH 8, and the protein was purified using Ni‐NTA affinity chromatography using a 5 mL HisTrap FF column (Cytiva) over a linear gradient of 20 mM–1 M imidazole for 10 CVs. Peak fractions containing NSP2 (as determined by SDS‐PAGE) were combined and diluted 1:1 with 20 mM HEPES‐Na pH 7.4. The protein was simultaneously concentrated and buffer exchanged to remove residual imidazole using centrifugal ultrafiltration (Sartorius Vivaspin 50,000 MWCO). The concentrated sample was further purified by size exclusion chromatography on a HiLoad 26/600 Superdex 200pg column (Cytiva) that was pre‐equilibrated with RNase‐free SEC buffer (20 mM HEPES, pH 7.4, 150 mM NaCl).

### 4.2 Fluorescence microscopy

In vitro images were recorded using an ONI Nanoimager S equipped with a 100× 1.4 NA oil immersion objective. N‐terminally tagged NSP5 and C‐terminally tagged NSP2 (pre‐incubated with 0.5 μM NTA‐Atto‐488) were mixed in equilibration buffer (1× PBS pH 7.4) or label buffer (1× PBS pH 6.6 in 100% D_2_O at 4°C) to give a final concentration of 1.25 μM of each protein or in quench buffer (PBS pH 1.8 in 0.1% DDM at 1°C) to give a final concentration of 0.75 μM. 4 μL of either sample was transferred to a glass cover slip and mounted on the Nanoimager. Fluorescent signal was detected by excitation of the sample with a 488 nm laser at a laser power of 2 mW. An area of 50μm × 80μm was recorded after 30 s, 2 min, 5 min, and 10 min incubation of the sample on the glass cover slip. ImageJ (Baggett et al., 2022) was used for automated particle counting and image analysis, and binary images were created by using Yen's thresholding method (Yen et al., 1995).

### 4.3 Native mass spectrometry

Native MS experiments were conducted on a Q‐Exactive Quadrupole Orbitrap UHMR Mass Spectrometer (Thermo Fisher) using in‐house prepared gold‐ and palladium‐coated nESI capillaries. Full‐length NSP5 was buffer exchanged into 200 mM ammonium acetate pH 6.9 and NSP5‐ΔCTR was buffer exchanged into 200 mM ammonium acetate pH 5.5 using 7 K MWCO Zeba spin desalting columns (Thermo Fisher) and diluted to a final monomeric concentration of 20 and 40 μM, respectively. Instrument parameters were set to capillary voltage = 1.5 kV, desolvation voltage = −150, resolution = 6000.

### 4.4 Hydrogen–deuterium exchange mass spectrometry (HDX‐MS)

HDX‐MS experiments were performed on an automated HDX robot (LEAP Technologies, USA) coupled to an Acquity M‐class LC and HDX Manager (Waters Corporation, UK). For NSP2 + NSP5 and NSP2 + NSP5‐ΔCTR, a total of 5 μL of protein containing solution containing NSP2 (25 μM), NSP5 / NSP5Δ‐CTR (25 μM), or a mixture of both (25 μM each) was added to 95 μL of deuterated buffer (1× PBS pD 7, 95% D_2_O). Samples were incubated at 4°C for 0, 0.5, 5, and 10 min. The exchange reaction was quenched post labeling by addition of 75 μL of quench buffer (1× PBS pH 1.8, 0.1% DDM) to 75 μL of the sample. For differential HDX‐MS of NSP5 alone to identify regions of partial structure, 5 μL of NSP5 at 10 μM was added to 95 μL of a deuterated buffer containing 25 mM potassium phosphate, 25 mM dipotassium phosphate, 300 mM NaCl in 85.5% D_2_O (pD 7) for either 30 s or 16 h. After labeling, HDX was quenched by adding 100 μL of quench buffer containing 25 mM potassium phosphate, 25 mM dipotassium phosphate, and 0.5% (w/v) DDM (pH 1.8) in 100 mL H_2_O. A total of 90 μL quenched sample was passed through an immobilized pepsin column (Enzymate, Waters Corporation, UK) and the resultant peptides were trapped on a VanGuard Pre‐Column (Acquity UPLC BEH C18 [17 μm, 2.1 mm × 5 mm], Waters Corporation, UK) for 3 min. Separation of peptide fragments was achieved with a C18 column (75 μm × 150 mm, Waters Corporation, UK) eluting over a linear gradient of 0%–40% (v/v) acetonitrile (0.1%[v/v] formic acid) in H_2_O (0.3% [v/v] formic acid) over 7 min at 40 μL min^−1^. Peptide fragments were detected using a Synapt G2‐Si mass spectrometer (Waters Corporation, UK), operated in mobility‐assisted data independent analysis with dynamic range extension enabled (HDMSe).

### 4.5 HDX‐MS analysis

Data were analyzed using PLGS and DynamX software (Waters Corporation, UK). Pepsin was excluded from the analysis, and restrictions for peptides in DynamX were minimum intensity = 1000, maximum sequence length = 25, minimum products per amino acid = 0.3, max ppm error = 10, file threshold = 2/3 replicates. Deuteros was used to identify statistically significant protected and deprotected peptides and to generate Woods plots (Lau et al., 2019). Statistically significant peptides were identified using an applied confidence interval of 98% that takes into account the variance in the measured uptake values, the number of timepoint observations for the variance, the number of timepoints, and a critical value for a 98% confidence limit for a two‐tailed *t*‐test, as outlined previously (Lau et al., 2019). A summary table of HDX‐MS parameters, in accordance with community guidelines (Masson et al., 2019), can be found in Supplementary Table 1.

## AUTHOR CONTRIBUTIONS

**Alice Colyer:** Writing – original draft; writing – review and editing; conceptualization; data curation; formal analysis; investigation; methodology; validation; visualization. **Julia Acker:** Writing – review and editing; conceptualization; data curation; formal analysis; investigation; methodology; validation; visualization. **Alexander Borodavka:** Writing – review and editing; conceptualization; funding acquisition; methodology; project administration; resources; software; supervision. **Antonio N. Calabrese:** Supervision; resources; software; project administration; writing – review and editing; methodology; conceptualization; funding acquisition.

## FUNDING INFORMATION

A. C. acknowledges PhD funding from the University of Leeds. J. A. acknowledges funding from the Engineering and Physical Sciences Research Council (EPSRC) doctoral training partnership (DTP) [2597129]. A. N. C. acknowledges support from a Sir Henry Dale Fellowship jointly funded by Wellcome and the Royal Society (220628/Z/20/Z). A. N. C. acknowledges support of a Royal Society research grant (RGS\R2\222357). A. B. acknowledges support from a Sir Henry Dale Fellowship jointly funded by Wellcome and the Royal Society (213437/Z/18/Z), and funding from Wellcome (307249/Z/23/Z). Funding from BBSRC (BB/M012573/1) and Wellcome (208385/Z/17/Z) enabled the purchase of mass spectrometry equipment.

## CONFLICT OF INTEREST STATEMENT

The authors declare no competing financial interest.

## Supporting information


**Data S1.** Supporting Information.

## Data Availability

The HDX‐MS data have been deposited to the ProteomeXchange Consortium via the PRIDE partner repository with the dataset identifier PXD058097.
