# Supplementary material for: Uncovering protein conformational dynamics within two‐component viral biomolecular condensates
Source: Protein Sci. 2025 Jun 16;34(7):e70181. doi: 10.1002/pro.70181 (PMC12168090; doi:10.1002/pro.70181)
Supplement: Supplementary file 1 — Data S1. Supporting Information. [file PRO-34-e70181-s001.docx]

**Supporting information**

**Uncovering protein conformational dynamics within two-component viral biomolecular condensates**

**Alice Colyer^1^, Julia Acker^2^, Alexander Borodavka^2^*, Antonio N. Calabrese^1^***

**^1^ Astbury Centre for Structural Molecular Biology**, School of Molecular and Cellular Biology, Faculty of Biological Sciences, University of Leeds, LS2 9JT, Leeds, United Kingdom

**^2^ Department of Chemical Engineering and Biotechnology,** University of Cambridge, CB2 1QW, Cambridge, United Kingdom

***Correspondence to Alexander Borodavka:*** ab2677@cam.ac.uk (A. Borodavka) ***and Antonio N. Calabrese*:** a.calabrese@leeds.ac.uk (A.N. Calabrese)


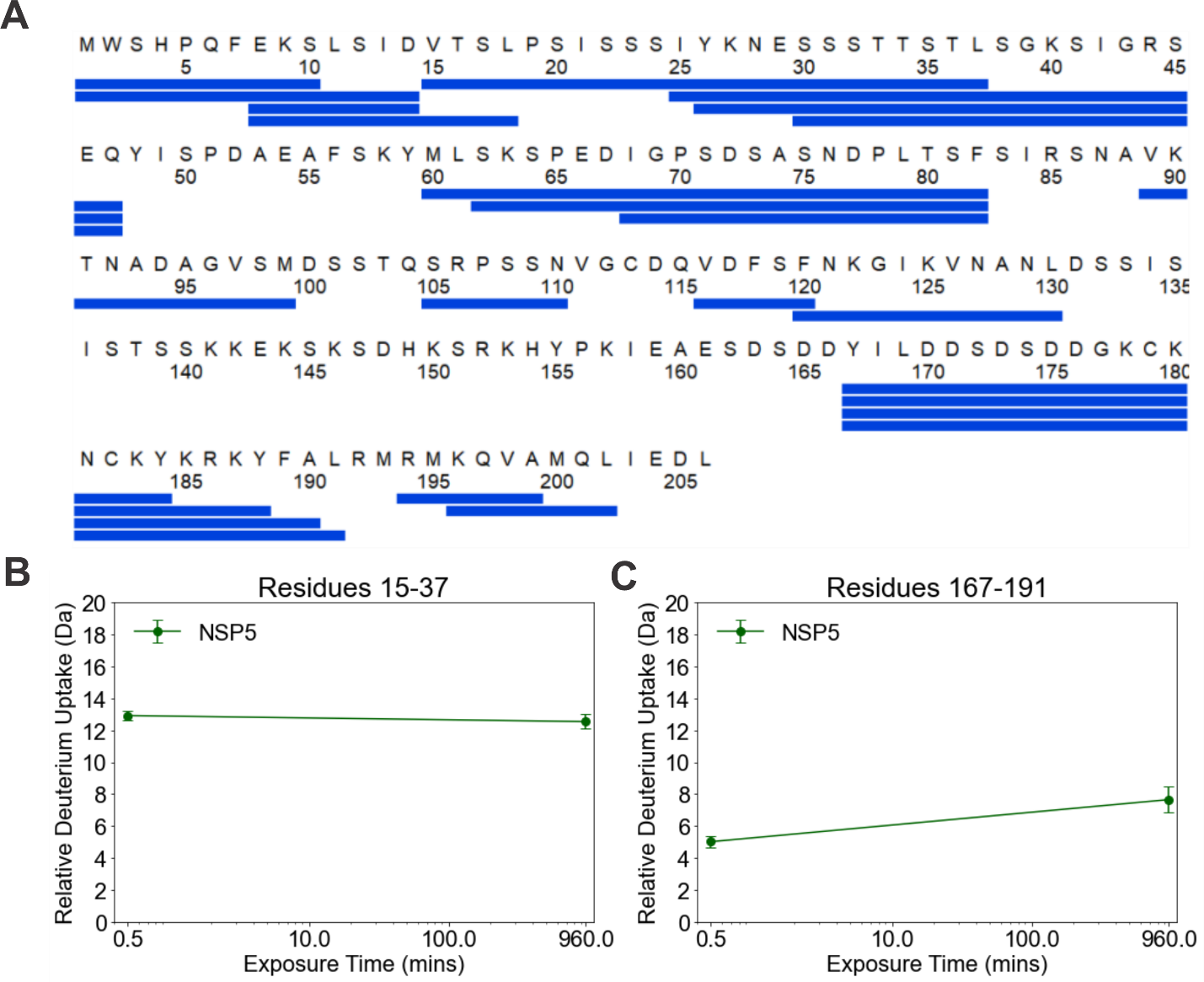


**Fig. S1** (A) Coverage map of differential HDX-MS of NSP5. Each blue bar represents an individual peptic peptide identified by HDX-MS. A total of 21 NSP5-derived peptides were identified after digestion with pepsin, representing over 66% sequence coverage. No coverage was observed between residues 131-166, likely due to the low sequence complexity of this region (which contains a high proportion of serine, lysine and aspartic acid residues that may not be cleaved by pepsin). (B) N-terminal peptide fragment appears to reach maximum exchange at the 30 second time point (residues 15-37), (C) whereas C-terminal peptide fragments appear to increase in relative deuterium uptake between 30 seconds and 16 hour time points (residues 167-191).

**Fig. S2** Number of condensates under equilibration, label and quench conditions across HDX-MS timepoints. Significant differences in the number of condensates were calculated by performing a one-way ANOVA (** = p≤ 0.001)

**
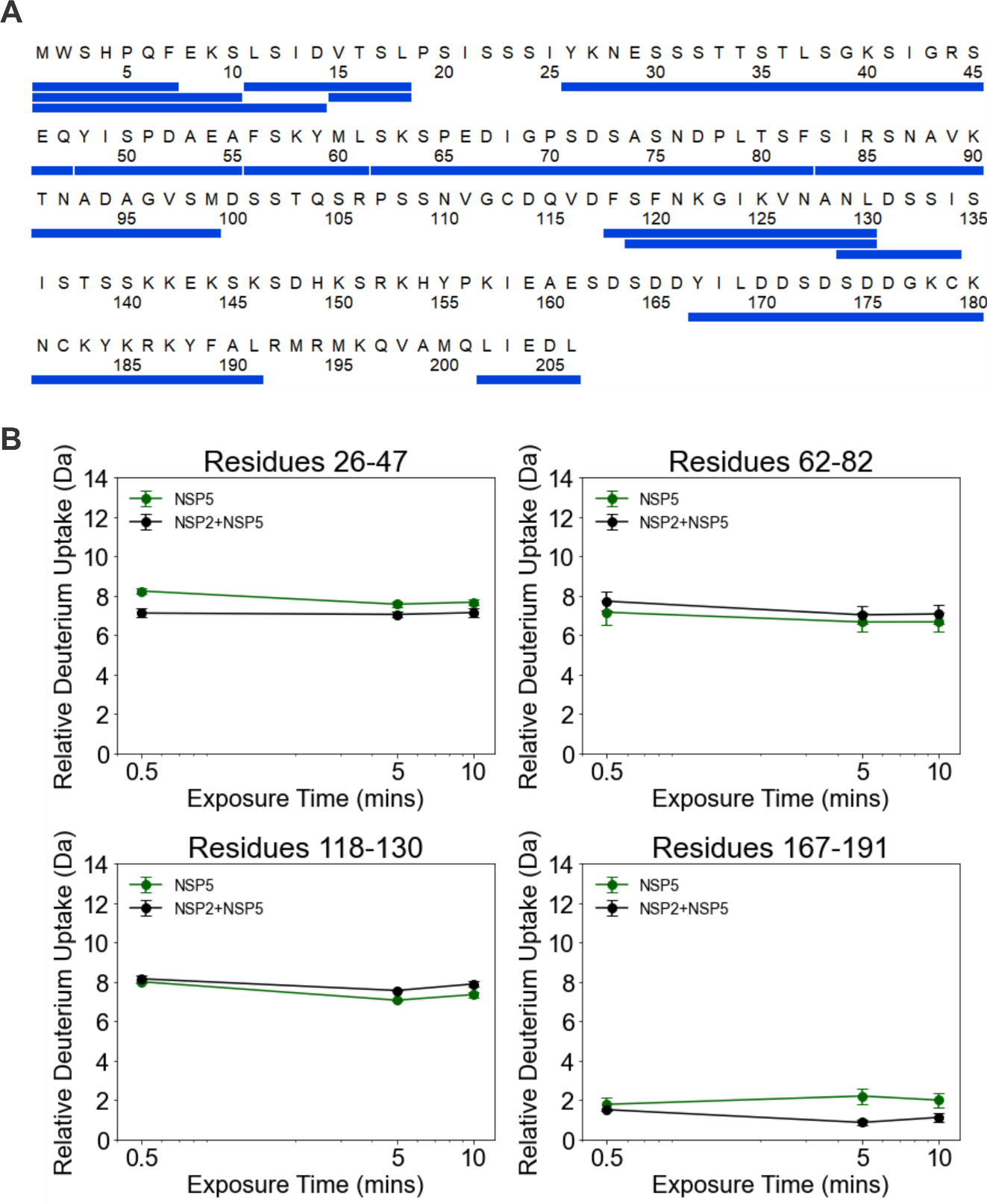
**

**Fig. S3** (A) Coverage map for HDX-MS of NSP5 in the presence of NSP2. Each blue bar represents an individual peptic peptide identified by HDX-MS. (B) Individual uptake plots for representative significantly protected and deprotected NSP5 peptides in the presence of NSP2. A small decrease in relative deuterium uptake is observed between 30 seconds and 5 minutes, which can occur due to reduced back exchange as a result of the LEAP robot, coupled to our mass spectrometry system, omitting a step when it is utilised for sample preparation for 2-minute time points or less (Lumpkin & Komives, 2019).

**
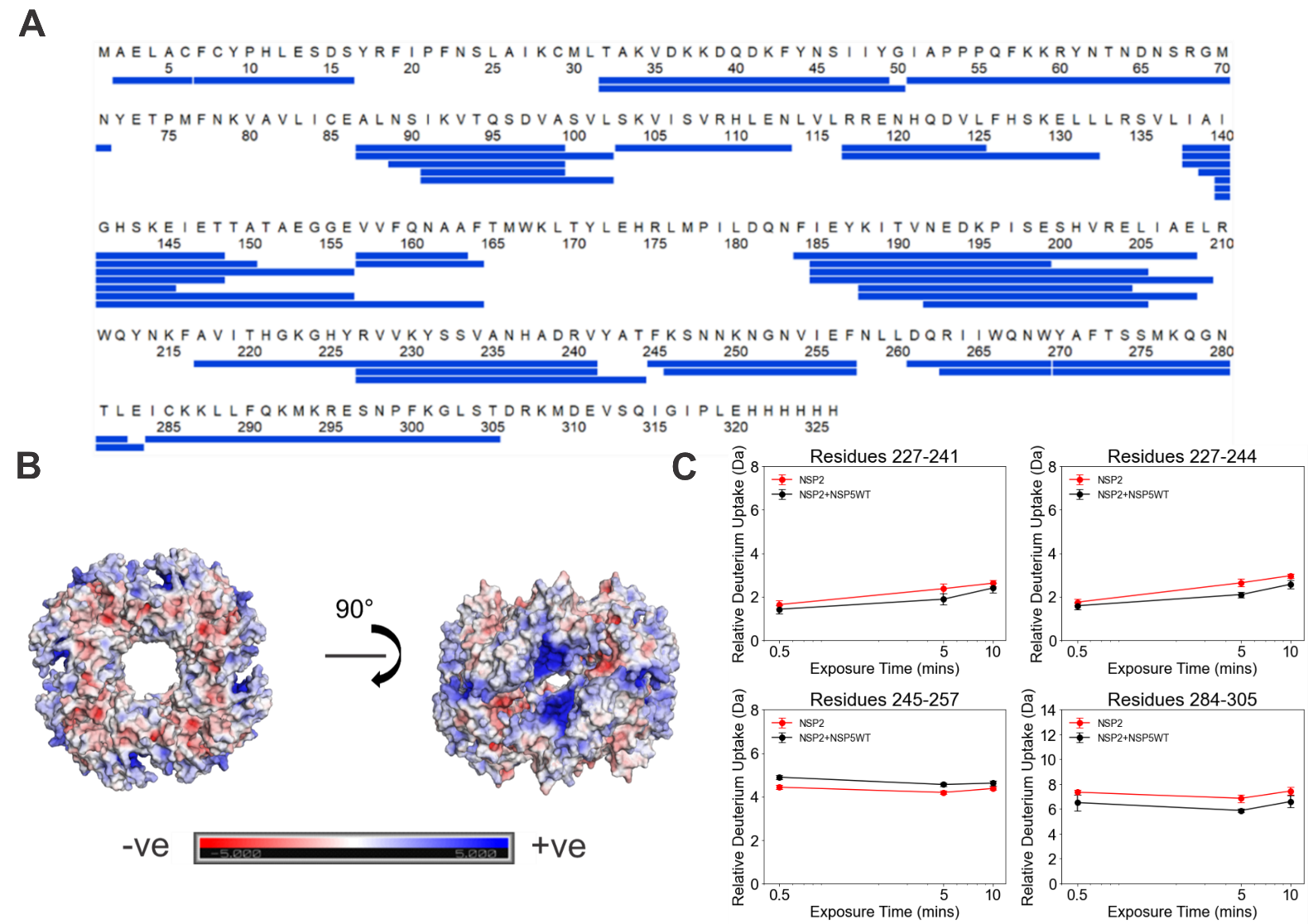
**

Fig. S4. (A) Coverage map for HDX-MS of NSP2 in the presence of NSP5. Each blue bar represents an individual peptic peptide identified by HDX-MS. (B) Electrostatic projection of NSP2 octamer rendered in Pymol (version 4.6) using APBS electrostatics plugin. (C) Individual uptake plots for protected peptides (residues 227-244 and 284-305) and deprotected peptides (residues 245-257). A small decrease in relative deuterium uptake is observed between 30 seconds and 5 minutes, which can occur due to reduced back exchange as a result of the LEAP robot, coupled to our mass spectrometry system, omitting a step when it is utilised for sample preparation for 2-minute time points or less (Lumpkin & Komives, 2019).


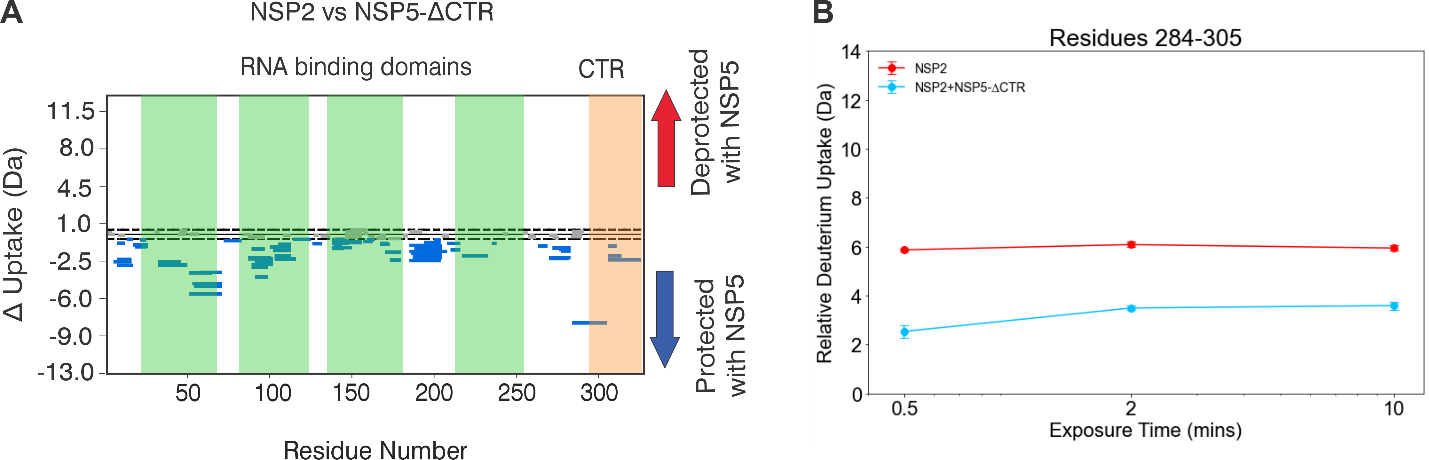


**Fig. S5.** (A) Cumulative Woods’ plot showing the summed differences in deuterium incorporation over all timepoints for NSP2 vs NSP5-ΔCTR. Peptides from NSP2 that were significantly protected and deprotected peptides when incubated with deuterium in the presence of NSP2 are shown in blue and red, respectively (hybrid statistical test, p < 0.02). (B) Individual uptake plot for the protected peptide spanning residues 284-305.

Table S1. HDX-MS summary data table

|  | **NSP5** | **NSP2 + NSP5** | **NSP5 + NSP2** | **NSP2 + NSP5-ΔCTR** |
| --- | --- | --- | --- | --- |
| HDX reaction details | 25mM potassium phosphate, 25mM dipotassium phosphate, 300mM NaCl in 85.5% D_2_O, pH 6.6, 4°C | 1X PBS in 95% D_2_O, pH 6.6, 4°C | | |
| HDX time points (mins) | 0.5, 960 | 0, 0.5, 5, 10 | | 0, 0.5, 2, 10 |
| HDX controls | Maximally labelled controls were not performed | | | |
| Back-exchange | N/A | N/A | N/A | N/A |
| No. of peptides | 21 | 39 | 15 | 122 |
| Sequence coverage | 66% | 72.7.% | 67.5% | 95.4% |
| Average peptide length / redundancy | 15.29 / 2.37 | 14.67 / 2.41 | 11.87 / 1.28 | 9.86 / 3.87 |
| Replicates | 3 (Technical) | 3 (Technical) | 3 (Technical) | 3 (Technical) |
| Repeatability | 0.098 (average SD) | 0.0595 (average SD) | 0.0787 (average SD) | 0.0354 (average SD) |
| Significant difference in HDX (delta HDX > XD) | p-value <0.05 | 98% CI: 0.26 Da / p-value <0.02 | 98% CI: 0.45 Da / p-value <0.02 | 98% CI: 0.17 Da / p-value 0.02 |

**References**

Lumpkin, R. J., & Komives, E. A. (2019). DECA, A Comprehensive, Automatic Post-processing Program for HDX-MS Data*. *Molecular & Cellular Proteomics*, *18*(12), 2516–2523. doi: 10.1074/mcp.TIR119.001731
